# The neurobiology of methamphetamine induced psychosis

**DOI:** 10.3389/fnhum.2014.00537

**Published:** 2014-07-22

**Authors:** Jennifer H. Hsieh, Dan J. Stein, Fleur M. Howells

**Affiliations:** Department of Psychiatry and Mental Health, University of Cape TownWestern Cape, South Africa

**Keywords:** schizophrenia, cortex, GABA, neurotoxicity, sensitization, neural circuitry

## Abstract

Chronic methamphetamine abuse commonly leads to psychosis, with positive and cognitive symptoms that are similar to those of schizophrenia. Methamphetamine induced psychosis (MAP) can persist and diagnoses of MAP often change to a diagnosis of schizophrenia over time. Studies in schizophrenia have found much evidence of cortical GABAergic dysfunction. Methamphetamine psychosis is a well studied model for schizophrenia, however there is little research on the effects of methamphetamine on cortical GABAergic function in the model, and the neurobiology of MAP is unknown. This paper reviews the effects of methamphetamine on dopaminergic pathways, with focus on its ability to increase glutamate release in the cortex. Excess cortical glutamate would likely damage GABAergic interneurons, and evidence of this disturbance as a result of methamphetamine treatment will be discussed. We propose that cortical GABAergic interneurons are particularly vulnerable to glutamate overflow as a result of subcellular location of NMDA receptors on interneurons in the cortex. Damage to cortical GABAergic function would lead to dysregulation of cortical signals, resulting in psychosis, and further support MAP as a model for schizophrenia.

## 1. Introduction

Methamphetamine is a lipophilic compound used recreationally for its ability to temporarily induce a variety of desirable effects, including increased energy levels, positive mood, euphoria, reduced appetite, weight loss, enhanced mental acuity, social, and sexual disinhibition (Cretzmeyer et al., 2003; Green and Halkitis, 2006; Cruickshank and Dyer, 2009).

According to the United Nations World Drug Report, between 0.3 and 1.3% of the world's population uses amphetamine-type stimulants (United Nations Office, 2011; Burns, 2014). Amphetamine abuse has a multitude of repercussions, including violence (Plüddemann et al., 2010) criminal behavior, incarceration, recidivism (Cartier et al., 2006), and the transmission of HIV (Halkitis et al., 2001; Colfax and Guzman, 2006).

Repeated administrations, or administration of high doses of methamphetamine, commonly leads to psychosis (Connell, 1958; Bell, 1965; Angrist and Gershon, 1970; Bell, 1973; Batki and Harris, 2004; Curran et al., 2004), where symptoms typically include paranoid delusions, auditory hallucinations, increased activity, and odd speech (Connell, 1958; Bramness et al., 2012). The prevalence of psychotic symptoms as a result of methamphetamine use can be difficult to determine as the diagnosis could be confused with that of a primary psychotic disorder, or other drug use (Weich and Pienaar, 2009; American Psychiatric Association, 2013). Depending on the study, the prevalence of psychosis in methamphetamine abusers ranges between 10 and 60% (Farrell et al., 2002; McKetin et al., 2006; Mahoney et al., 2008). In Thailand, approximately 10% of admissions to psychiatric facilities are attributed to methamphetamine related psychosis (Farrell et al., 2002). An Australian study of 277 non-treatment-seeking illicit methamphetamine users with no prior diagnosis of schizophrenia or other psychotic disorders found that 51 (18%) had “clinically significant” psychotic symptoms (McKetin et al., 2006), and a U.S. study of 42 cocaine-dependent and 43 methamphetamine-dependent individuals, screened to exclude patients with other axis I disorders, reported psychotic symptoms of at least some type in at least 60% of both groups (Mahoney et al., 2008). Recreational use of methamphetamine has been shown to increase the risk of developing psychotic symptoms by two to three fold (McKetin et al., 2010) especially if larger amounts of methamphetamine is used, or use began at a younger age (Chen et al., 2003).

Symptoms of MAP were originally thought to dissipate within a week of methamphetamine withdrawal (Connell, 1958). However, studies have shown that, although the large majority of MAP symptoms resolve within 1 month (Iwanami et al., 1994; Ujike and Sato, 2004; Deng et al., 2012), 30% of those with MAP had symptoms persist for up to 6 months (Deng et al., 2012), and 10–28% reported persisting symptoms for more than 6 months (Iwanami et al., 1994; Deng et al., 2012). Symptoms of MAP have been shown to relapse after long periods of abstinence (Sato et al., 1992; Yui et al., 1997, 1999).

The DSM-V indicates that persistent psychosis after 6 months of abstinence from methamphetamine can constitute a diagnosis of schizophrenia (American Psychiatric Association, 2013). A recent Chinese study found that 5% of patients initially diagnosed with MAP had their diagnosis changed to schizophrenia in the interview conducted by the study (Deng et al., 2012). In Thailand, a similar study found that 38.8% of abstinent methamphetamine abusers who were initially hospitalized for MAP, were subsequently given a diagnosis of schizophrenia (Kittirattanapaiboon et al., 2010).

Whether there are fewer presentations of negative symptoms (lack of affect, social withdrawal) in MAP than schizophrenia (Tomiyama, 1990; Panenka et al., 2013), or whether both positive and negative symptom presentations are similar in MAP and schizophrenia (Srisurapanont et al., 2003, 2011), is still under debate. However, there is broad consensus that the positive symptoms of psychosis induced by methamphetamine use are difficult to distinguish from the positive symptoms of schizophrenia (Connell, 1958; Janowsky and Risch, 1979; Bramness et al., 2012; Medhus et al., 2013).

Clinical similarities between MAP and schizophrenia have long since been recognized (Connell, 1958). Various methamphetamine induced animal models for psychosis such as the neurotoxicity model (Robinson and Becker, 1986; Machiyama, 1992; Davidson et al., 2001; Cadet and Krasnova, 2009), the behavioral sensitization model (Castner and Goldman-Rakic, 1999; Featherstone et al., 2007), or the escalating dose-binge model (Segal and Kuczenski, 1997, 2001), have been proposed and debated for validity. Most relevant to this review is the methamphetamine sensitization model, where lower doses of repeated methamphetamine exposure have been shown to produce behavioral effects that best model psychosis by measurements such as increased locomotion, hallucinatory behaviors in the case of non-human primates, and deficits in pre-pulse inhibition, latent inhibition, and other cognitive measures in a variety of animal models (Castner and Goldman-Rakic, 1999; Kamei et al., 2006; Featherstone et al., 2007; Nagai et al., 2007; Forrest et al., 2014).

Schizophrenia has been associated with a wide variety of neurocognitive deficits e.g., in verbal memory, social cognition, implicit learning, and working memory, which impair day to day function (Green, 1996; Gold, 2004; Horan et al., 2008). Cognitive functions such as working memory rely on coherent interaction between interaction between interneurons and pyramidal cells, which form local memory fields, and “sharpen” signal to noise ratios (Goldman-Rakic, 1995). GABAergic interneurons in the cortex shape the stimulus response properties of pyramidal cells, and prevent aberrant firing of cortical processes (Jones, 1993). Evidence for GABA dysfunction in schizophrenia is convincing, and has been reviewed extensively (Goldman-Rakic, 1994; Lewis et al., 2004; Daskalakis et al., 2007; Lewis et al., 2012).

If MAP is to be a good model for schizophrenia, it should also exhibit similar cognitive impairments, and similar evidence of GABA disturbances as schizophrenia. In humans, MAP presents with similar cognitive impairments as those in schizophrenia, such as deficits in verbal and working memory, as well as other executive functions (Scott et al., 2007; Jacobs et al., 2008). In animal models, cognitive impairments have been found in using the amphetamine sensitization model in the non-human primate (Castner et al., 2005), and rodents (Nagai et al., 2007; Arai et al., 2009). Indeed, emerging evidence that will be discussed in this paper suggests that the cognitive deficits in MAP may also result from a GABAergic dysfunction in the cortex, although the mechanism for these observations are unknown.

The present paper will first describe the well characterized and relevant nigrostriatal, mesolimbic, and mesocortical pathways, on which methamphetamine exerts its major effects. Second, we will review the acute effects of methamphetamine and chronic effects of methamphetamine on these pathways. Next, studies that have shown cortical GABAergic disturbance will be discussed. Finally, we propose a possible vulnerability of GABAergic interneurons in the cortex to glutamate overflow, which might explain the GABAergic disturbance by methamphetamine that might lead to psychosis.

## 2. Neurocircuitry of the cortex and basal ganglia

**Figure 1:**
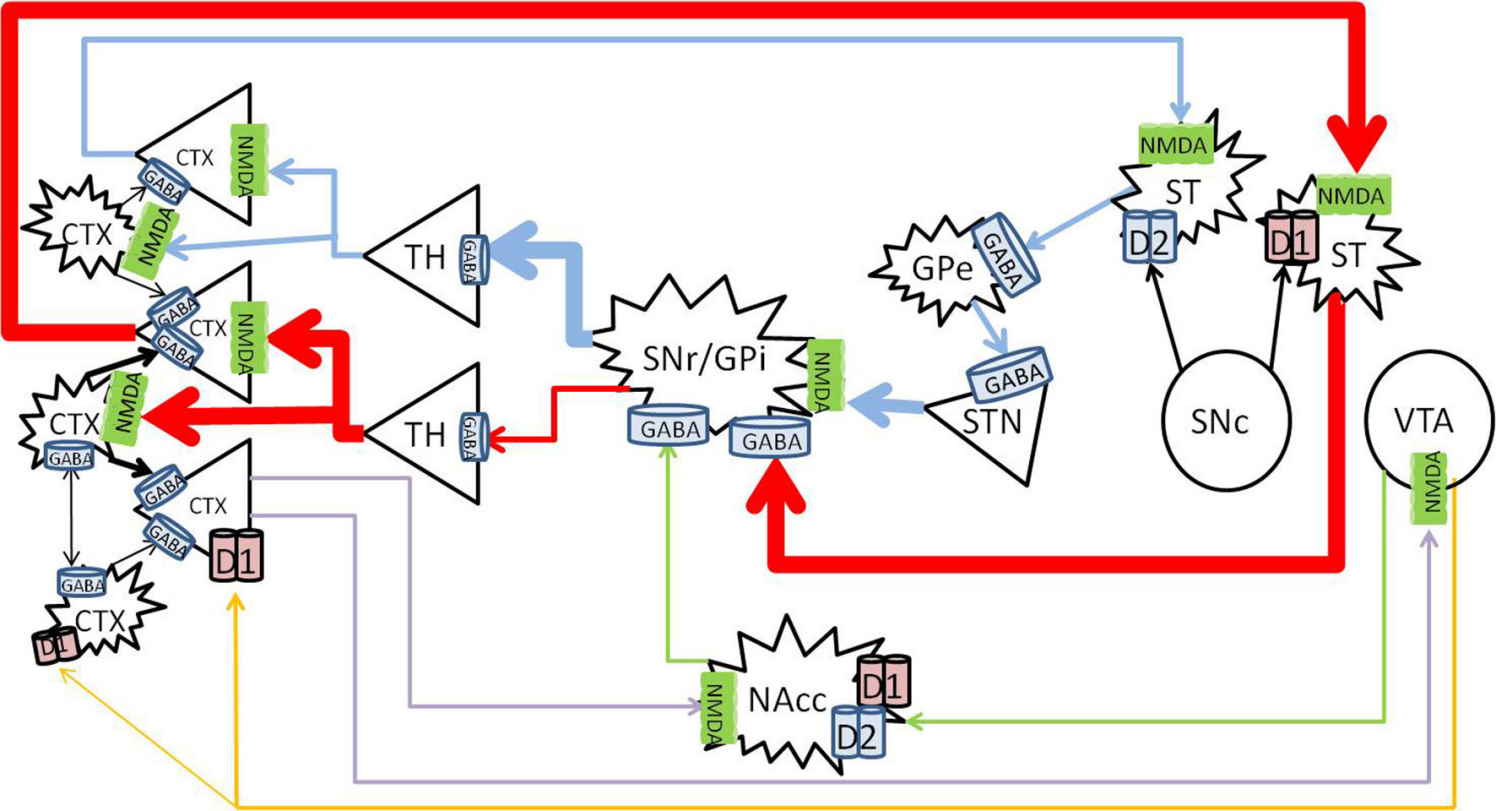
**Nigrostriatal, mesocortical, and mesolimbic pathways**. When cortical neurons activate the NMDA receptors on neurons of the direct pathway (*red*) in the striatum, striatal neurons are primed to send GABA signals to the SNr/GPi, which would inhibit the tonic GABA to the thalamus, allow glutamate signals to fire in the cortex, and further activate cortical neurons. The indirect pathway (*blue*) is primed when cortical neurons activate the NMDA receptors on the striatal neurons that express D_2_ receptors, which would send inhibitory signals to the globus pallidus externa (GPe), reduce GABA signaling to the STN, and stimulate the SNr/GPi to enhance GABA inhibiting of thalamocortical signaling. The mesolimbic pathway (*green*) consist of dopaminergic projections from the VTA to the NAcc, which sends inhibitory signals to the SNr/GPi. The mesocortical pathway (*orange*) consists of dopaminergic projections from the VTA to the cortex, innervating both pyramidal and non-pyramidal neurons which express D_1_ receptors. Reciprocal pathways (purple) from the cortex to the VTA and NAcc provide cortical feedback to subcortical structures. CTX, cortex; GABA, gamma-aminobutyric acid; NMDA, N-methyl-D-aspartate; GPe, globus pallidus externa; SNr, substantia nigra pars reticulata; GPi, globus pallidus interna; TH, thalamus; STN, sub thalamic nucleus; NAcc, nucleus accumbens core; VTA, ventral tegmental area. Round structures indicate dopamine cell bodies; star shaped structures indicate GABA cell bodies; triangular structures indicate glutamate cell bodies.

Methamphetamine appears to primarily affect the dopaminergic system. The present review will focus on the three main pathways, namely the nigrostriatal, mesolimbic, and mesocortical pathways, summarized in Figure 1.

The nigrostriatal pathway consists of the known direct and indirect pathways, which control the expression and direction of behavior to predictable stimuli or rewards (Balleine et al., 2007; Nicola, 2007). The mesolimbic pathway is involved in reward processing (Koob and Bloom, 1988), effort related functions (Salamone et al., 2007), translation of emotions into actions (Mogenson et al., 1980), and direction of behavior where stimuli and rewards are less predictable (Nicola, 2007). The mesocortical pathway is involved with cognitive functions such as working memory (Moal, 1980; Goldman-Rakic et al., 2004).

The majority (up to 77%) of the striatum consists of GABAergic projection neurons (Graveland et al., 1985), that receive topographical input from the cortex (Kemp and Powell, 1970; Alexander and Crutcher, 1990; Haber and Knutson, 2010), and express *N*-methyl-*D*-aspartate (NMDA) receptors (Albin et al., 1992). The projection neurons comprises two major sub-populations. The striatonigral neurons, characterized by the expression of D_1_ receptors, project to the basal ganglial output nuclei, substantia nigra pars reticulata (SNr)/globus pallidus interna (GPi) (Altar and Hauser, 1987), the starting point of the direct pathway. The striatopallidal neurons, characterized by the expression of D_2_ receptors, project to the globus pallidus externa (GPe), and begin the indirect pathway. In the indirect pathway, neurons in the (GPe) send GABAergic projections to the subthalamic nucleus (STN), which send glutamatergic projections to activate the SNr/GPi. The SNr consists of tonically active GABAergic neurons that project to the thalamus (Reubi et al., 1977, 1978), and the thalamus projects via topographical glutamatergic neurons to the cortex (Haber and Knutson, 2010).

Since the SNr GABAergic neurons are tonically active (Reubi et al., 1977, 1978), there is normally little movement at rest, or minimal signaling to the cortex from the thalamus. In addition, up to 23% of striatal neurons are interneurons which have inhibitory effects on the projection neurons (Rymar et al., 2004), further inhibiting thalamocortical signals at rest (Reubi et al., 1977, 1978). Cortical glutamatergic activation of either the direct or indirect pathway primes the relevant pathway to an “upstate” (Wilson and Kawaguchi, 1996). From this “upstate,” a small additional increase in activation by dopamine, released from the SNc, or a small decrease in inhibition by striatal cholinergic interneurons, will lead to the generation of action potentials that finally activate the primed pathway (Wilson, 1993; Tepper and Bolam, 2004).

**Figure 2:**
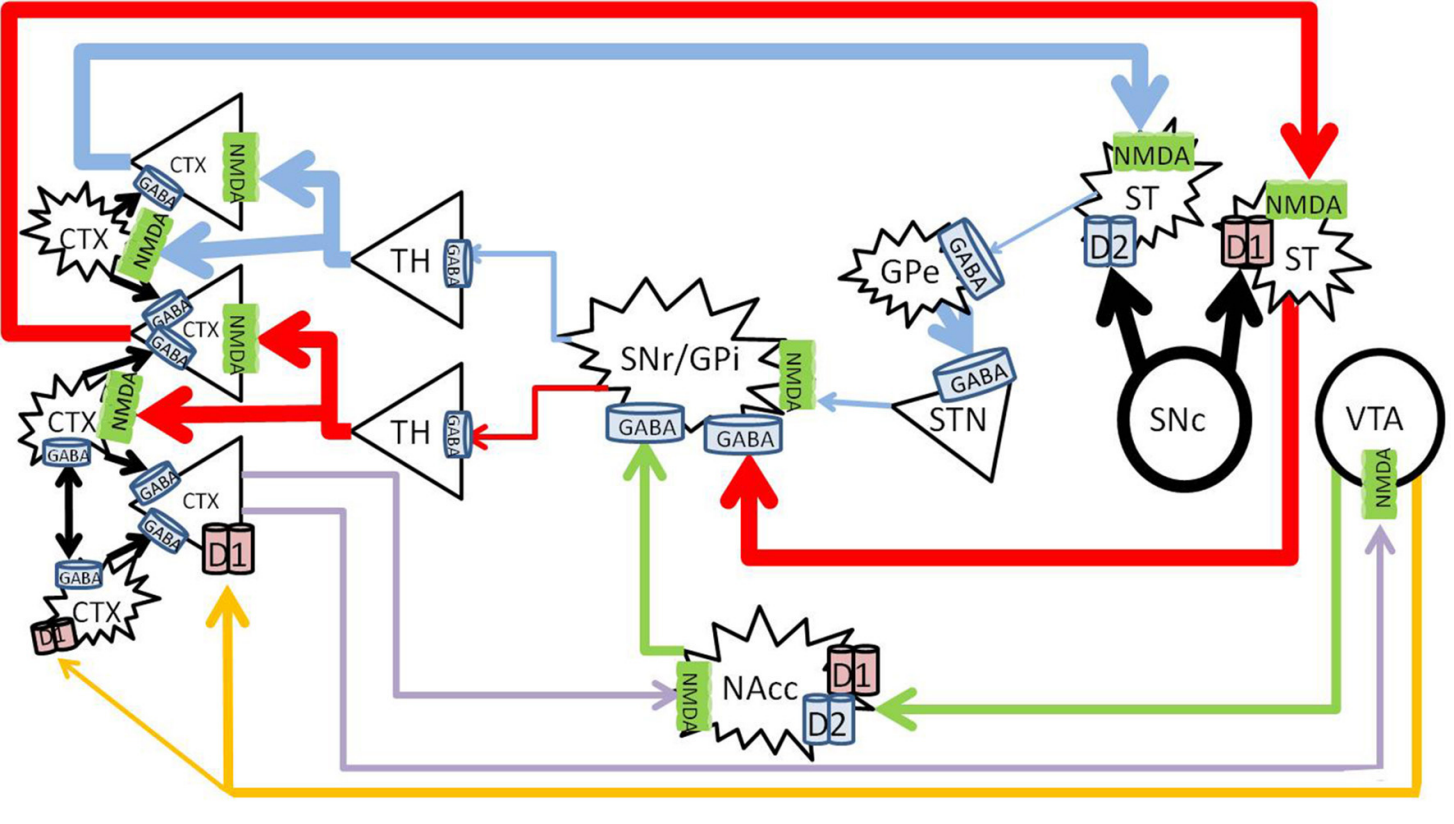
**Healthy effects of dopamine on dopaminergic pathways**. The substantia nigra pars compacta (SNc) releases dopamine into the striatum (large black arrows), and enhances thalamocortical glutamate release along the nigrostriatal pathway (*blue and red*). The direct pathway (*red*) becomes enhanced by D_1_ activation, and the indirect pathway (*blue*) becomes inhibited by D_2_ activation. Mesolimbic dopamine (*green*) to the NAc core increases GABA inhibition from the NAc core to the SNr/GPi, further increasing thalamocortical glutamate transmission. Mesocortical dopamine (*orange*) increases cortical pyramidal firing and simultaneously stimulates cortical interneurons, which sharpen the pyramidal signals to subcortical nuclei (*blue, red, and purple*). CTX, cortex; GABA, gamma-aminobutyric acid; NMDA, N-methyl-D-aspartate; GPe, globus pallidus externa; SNr, substantia nigra pars reticulata; GPi, globus pallidus interna; TH, thalamus; STN, sub thalamic nucleus; NAcc, nucleus accumbens core; VTA, ventral tegmental area. Round structures indicate dopamine cell bodies; star shaped structures indicate GABA cell bodies; triangular structures indicate glutamate cell bodies.

Activation of the dopamine system enhances glutamate signaling in the cortex from the nigrostriatal and mesolimbic pathways, and increases dopamine in the prefrontal cortex from the mesocortical pathway (Figure 2).

Dopamine causes activation of neurons expressing D_1_ receptors, and inhibits neurons which express D_2_ receptors (Surmeier et al., 2007). In the nigrostriatal pathway, the synergistic activation of both the D_1_ and D_2_ receptors allows dopamine to enhance glutamate induced firing in the striatum (Hu and White, 1997). Thus, dopamine release into the striatum from the SNc enhances thalamocortical signals by simultaneously activating the direct pathway, and inhibiting the indirect pathway. Long term potentiation from this glutamatergic transmission leads to learning of the relevant activity, and the development of persistent behaviors (Yin and Knowlton, 2006; Balleine et al., 2007).

The mesolimbic pathway arises from dopaminergic ventral tegmental area (VTA) projections to the nucleus accumbens (NAc) (Fallon and Moore, 1978). The efferent projections of the NAc and afferent connections with other neuronal structures such as the amygdala, hippocampus and cortex are complex, and mediate its role in direction of behavior to emotive stimuli (Haber and McFarland, 1999; Haber and Knutson, 2010). Briefly, the NAc consists of “core” and “shell” regions, and one of the major effects of dopamine release at the NAc core is to increase GABA release to the SNr/GPi, disinhibiting the thalamus and increasing cortical glutamate (Maurice et al., 1999; Nicola, 2007).

The VTA and SN project to the cortex along the mesocortical pathway, and terminates on both pyramidal and non-pyramidal cells in the pre-frontal cortex (PFC) (Goldman-Rakic et al., 1989; Williams and Goldman-Rakic, 1998; Tzschentke, 2001). The expression of D_1_ receptors is higher than that of D_2_ in the cortex (Gaspar et al., 1995), and although more prevalent on pyramidal neurons, D_1_ receptors are also widely expressed on GABAergic interneurons (Muly et al., 1998). Dopamine in the PFC serves a modulatory purpose and enhances D_1_ associated firing (Cepeda and Levine, 1998). Optimal levels of dopamine stimulates cortical GABAergic interneurons, which “tune” the pyramidal firing to sharpen pyramidal signals and enhances cognitive performance (Muly et al., 1998).

Cortical interneurons are predominantly GABAergic and prevent aberrant firing of cortical processes by shaping the receptive fields of thalamocorical signals (Jones, 1993). In addition, interneurons facilitate feed-forward inhibition, where a single thamalmic fiber can activate its pyramidal target as well as an interneuron, which will inhibit the pyramidal target within 1–10 ms, providing fine temporal control of pyramidal firing (Gabernet et al., 2005). These cells are necessary for inhibitory modulation, disinhibitory modulation, discriminative processing, gating of signals, as well as contribute in the generation of oscillatory rhythms that unify pyramidal cell discharge (Benes and Berretta, 2001).

## 3. Acute effects of methamphetamine

**Figure 3:**
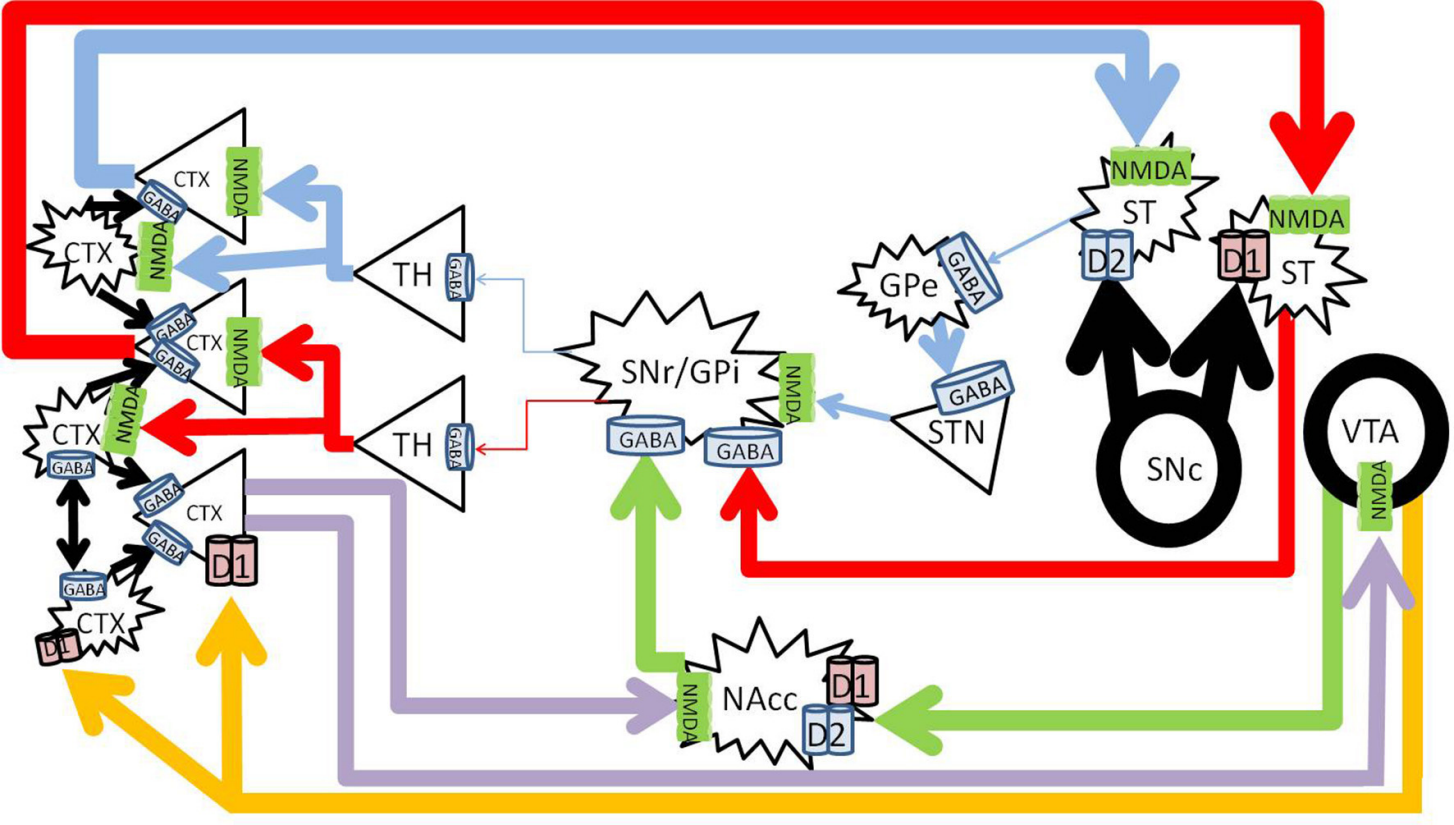
**Methamphetamine effects on dopaminergic pathways**. Methamphetamine causes excessive amounts of dopamine to be released from the substantia nigra pars compacta (SNc) into the striatum (large black arrows), forcing pronounced inhibition of the SNr/GPi (*red and blue*). Dopamine from the VTA to the NAc increases NAc inhibition on the SNr/GPi (*green*). Enhanced cortical signals increases glutamate from the PFC to the NAc (*purple*) further increasing NAc inhibition on the SNr/GPi, and exacerbates glutamate excess in the cortex. CTX, cortex; GABA, gamma-aminobutyric acid; NMDA, N-methyl-D-aspartate; GPe, globus pallidus externa; SNr, substantia nigra pars reticulata; GPi, globus pallidus interna; TH, thalamus; STN, sub thalamic nucleus; NAcc, nucleus accumbens core; VTA, ventral tegmental area. Round structures indicate dopamine cell bodies; star shaped structures indicate GABA cell bodies; triangular structures indicate glutamate cell bodies.

Acute methamphetamine administration causes vesicular release of dopamine from the VTA into the NAc and PFC in the mesolimbic and mesocortical pathways (Fallon and Moore, 1978; Haber and Knutson, 2010). Methamphetamine also reverses both vesicular monoamine transporter 2 and the dopamine transporter (Sulzer et al., 1995; Sora et al., 2009) to effectively increase synaptic concentrations of dopamine in the striatum in the nigrostriatal pathway (Bustamante et al., 2002; Fowler et al., 2008; Haber and Knutson, 2010) (Figure 3).

Acutely, methamphetamine increases levels of glutamate in the striatum (Stephans and Yamamoto, 1994) and the PFC (Stephans and Yamamoto, 1995). This is achieved via a polysynaptic process, where the increased dopamine release activates gamma-aminobutyric acid (GABA) neurons in the striatum of the direct pathway and increases GABA release at their terminals in the SNr. Pre-synaptic D_1_ receptors on the striatonigral terminals (Altar and Hauser, 1987) also enhance GABA release at the SNr (Mark et al., 2004). The increased GABA at the SNr cell bodies disinhibit the thalamocortical glutamatergic pathway (Mark et al., 2004), which results in an increase of glutamate within the cortex (Timmerman and Westerink, 1997). The high levels of glutamate at the level of the cortex activates the glutamatergic corticostriatal neurons via topographically specific postsynaptic density connections (Gerfen, 1989; Bellomo et al., 1998), and increases glutamate release in the striatum (Stephans and Yamamoto, 1994), driving positive feedback.

Glutamate levels in the striatum in fact remain elevated for over 28 h, even after dopamine has returned to basal levels (Mark et al., 2004, 2007). It was found that the vesicular glutamate transporter 1 expression was increased, which allows for the sequestration of glutamate in the cortex, and thus allowing extended glutamate release into the striatum after methamphetamine administration (Mark et al., 2007). The NMDA receptors in the striatum facilitate D_1_ receptor mediated currents, which positively drives this circuit.

Acute methamphetamine administration has been found to decrease GABA_B_ transmission within the VTA (Padgett et al., 2012), and increases dopamine release from the VTA to the NAc (Fallon and Moore, 1978; Kankaanpää et al., 1998; Zhang et al., 2001). This release of dopamine in the mesolimbic pathway is associated with the experience of reward or euphoria (Volkow et al., 1996; Drevets et al., 2001), and increases subsequent GABA release to the substantia nigra for longer than 80 min (Sirinathsinghji et al., 1988). Inhibition of the SNr disinhibits the thalamocortical glutamatergic pathway, and results in increased cortical glutamate (Mark et al., 2004).

Methamphetamine also increases dopamine release from the VTA to the prefrontal cortex in the mesocortical pathway (Fallon and Moore, 1978; Stephans and Yamamoto, 1995). At low doses, methamphetamine can increase cognitive performance by activation of D_1_ receptors in the PFC (Silber et al., 2006), however high doses impair cognitive function (Schroder et al., 2003), possibly by over activation of D_1_ receptors on interneurons in the PFC and excessive inhibition of cortical signals (Muly et al., 1998).

Methamphetamine can thus increase glutamatergic signals to the cortex from both the nigrostriatal, as well as the mesolimbic reward circuits, and increase dopaminergic signals from the mesocortical pathway. Excessive glutamate and dopamine in the cortex may overwhelm GABAergic interneuons, causing dysregulation of the signals, which may relate to the psychotic symptoms that can occur during methamphetamine intoxication in some individuals.

## 4. Chronic methamphetamine effects

Studies in chronic methamphetamine abusers have found several changes in the brain, particularly within the striatum. A post mortem study found decreased levels of dopamine terminal markers such as dopamine, tyrosine hydroxylase, and dopamine transporter in the striatum (Wilson et al., 1996). Positron emission tomography research found decreased levels of the dopamine transporter (Volkow et al., 2001b), decreased D_2_ (Volkow et al., 2001a) and decreased vesicular monoamine transporter 2 in the striatum (Chang et al., 2007). Single photon emission computed tomography studies have found decreased regional cerebral blood flow in chronic methamphetamine abusers (Iyo et al., 1997; Chung et al., 2010), magnetic resonance spectroscopy found decreased *N*-acetylaspartate (NAA) in the basal ganglia and frontal lobe (Ernst et al., 2000; Howells et al., 2014), and anterior cingulate (Nordahl et al., 2002; Howells et al., 2014) indicative of decreased neuronal integrity in these areas.

Participants with MAP have shown decreased P300 amplitude and increased P300 latency, particularly over the frontal cortex in electroencephalography (EEG) event related potential (ERP) studies, which suggests dysfunctional higher cognitive processing (Iwanami et al., 1991, 1993, 1998). Magnetic resonance spectroscopy showed decreased creatine and phosphocreatine to choline (Cr+PCr/Cho) ratio in the basal ganglia that correlated significantly with increased residual psychiatric symptom severity (Sekine et al., 2002). Magnetic resonance imaging processed for voxel based morphometry found decreased frontopolar volumes compared to healthy controls (Aoki et al., 2013).

Various regimen of methamphetamine administration have developed different models for MAP and schizophrenia. The neurotoxicity model typically gave high doses of methamphetamine in a short amount of time, and found extensive neurotoxic damage, particularly to the nigrostriatal and mesolimbic systems (Robinson and Becker, 1986; Machiyama, 1992; Davidson et al., 2001; Cadet and Krasnova, 2009). However, it has been argued that the severe neurotoxicity produced by this model is not similar to the lack of obvious neurotoxic damage in people with MAP and schizophrenia, in addition methamphetamine abusers tend to begin with low doses of methamphetamine, which have shown to be neuroprotective to subsequent higher doses. Most relevant to this review is the methamphetamine sensitization model, where lower doses of repeated methamphetamine exposure have been shown to produce behavioral effects that best model psychosis by measurements such as increased locomotion, hallucinatory behaviors in the case of non-human primates, pre-pulse inhibition deficits, latent inhibition deficits, other cognitive deficits (Castner and Goldman-Rakic, 1999; Castner et al., 2000; Kamei et al., 2006; Featherstone et al., 2007; Nagai et al., 2007; Forrest et al., 2014). Although, the escalating dose-binge model also appears to model a widely used pattern of drug taking by abusers (Segal and Kuczenski, 1997, 2001).

Methamphetamine sensitized animals showed increased glutamate levels in the VTA 3 days after withdrawal (Giorgetti et al., 2002). The enhanced VTA activation increased release of dopamine in the NAc (Gonon, 1988) and dorsal striatum with a low dose amphetamine challenge after 28 days of withdrawal (Paulson and Robinson, 1995). Glutamate release was found to be increased in the NAc 2 days post withdrawal with low dose amphetamine challenge (Xue et al., 1996), or with K^+^ depolarization 1 day post withdrawal (Bustamante et al., 2002). Studies in addiction have found that the increased sensitivity of the NAc to glutamate, and the increased glutamate release from the PFC to the NAc, mediates craving, and relapse in response to drug-related cues (Cornish and Kalivas, 2000; Kalivas and Volkow, 2005). Hyper-sensitivity of the NAc would further increase the activation of the NAc-thalamocortical circuit, and increase cortical glutamate (Maurice et al., 1999; Nicola, 2007).

Methamphetamine withdrawal after sensitization also exaggerated the inhibitory responses in the medial PFC, while augmenting excitatory responses in the ocular frontal cortex (Homayoun and Moghaddam, 2006). Morphological studies have found increased dendritic branching in the NAc and PFC of rodents approximately 1 month after methamphetamine sensitization (Robinson and Kolb, 1997, 1999), while a study in the dorsolateral PFC of non-human primates found decreased dendritic length and branching approximately 3 years after methamphetamine sensitization (Selemon et al., 2007). Twelve weeks of repeated low dose amphetamine exposure in rhesus monkeys showed reduced [123]IBZM displacement, and thus reduced dopamine release, in the striatum in response to a low dose amphetamine challenge about 30 days post withdrawal (Castner et al., 2000). Work on non-human primates has also shown that the cognitive deficits produced by methamphetamine sensitization are related to reduced dopaminergic turnover in the PFC and striatum (Castner et al., 2005), mediated by the D1-ERK pathway dysfunction in the PFC (Nagai and Yamada, 2010).

While studies in the methamphetamine sensitization model of psychosis is extensive (Featherstone et al., 2007), few have examined the effects of chronic methamphetamine exposure on the cortical GABAergic system.

## 5. Chronic methamphetamine effects on cortical GABA

Little research using any model has studied methamphetamine effects on GABA in the cortex, however there is some evidence which indicates that methamphetamine may indeed cause damage to cortical GABAergic interneurons.

Postmortem staining of the cortex in HIV positive methamphetamine users, compared to HIV positive non-users found extensive loss of calbindin immunoreactive interneurons in the cortex (Langford et al., 2003). Although co-morbid HIV and methamphetamine is known to compound neurodegenerative effects (Liu et al., 2009; Reiner et al., 2009), another group also found decreased calbindin immunoreactivity in cortical interneurons in rats that underwent an escalating dose, multiple binge exposure to methamphetamine, without the HIV confound (Kuczenski et al., 2007).

The escalating dose, multiple binge exposure of methamphetamine to the rats (0.1 mg/kg escalated in 0.1 mg/kg increments 3 times a day, to 4 mg/kg over 14 days, then 4 injections of 6 mg/kg every 2 h for 11 successive days) in the Kuczenski et al. (2007) study deserves further discussion. The loss of calbindin immunoreactive interneurons was apparent from 3 days after last exposure to methamphetamine, and persisted when examined at 30 days post exposure. Pyramidal neurons and dendrites (marked by NeuN and MAP2 staining, respectively), were not significantly lost at 3 days post exposure, but were significantly reduced at 30 days post exposure, particularly in layers 2, 3, and 5 (Kuczenski et al., 2007). These results suggest a particular vulnerability of interneurons to methamphetamine administration, as compared to pyramidal neurons. The contrary observation of loss of pyramidal cells and dendrites in this study and the increased dendritic branching and spine densities in the PFC and NAc as seen in earlier studies (Robinson and Kolb, 1997, 1999) was not discussed. The Kuczenski study appears to be supported by the morphological study in non-human primates that showed decreased dendritic branching in the dorsolateral PFC 3 years post amphetamine sensitization (Selemon et al., 2007). Selemon et al. explained possible discrepancies between the primate study and Robinson's studies to species differences or interval of methamphetamine discontinuation, however the two rodent studies were both performed in Sprague-Dawley rats, and examined at 30 (Kuczenski et al., 2007) or 38 (Robinson and Kolb, 1997, 1999) days post exposure. In addition, the methamphetamine administration regimen was similar, where the Robinson and Kolb study escalated doses of *D*-amphetamine from 1 to 8 mg/kg for 5 weeks excluding weekends, and gave 8 mg/kg doses twice a day, 4 h apart for the last 4 days (Robinson and Kolb, 1997). Further work regarding methamphetamine effects on cortical morphometry may be helpful to clarify these discrepancies.

A toxicity study delivered a continuous high dose (32 mg/kg/day) of methamphetamine by implanted minipump in rats for 5 days (Armstrong and Noguchi, 2004). Autoradiography of GABA_A_ sites labeled with [^3^H]-Flunitrazepam showed that methamphetamine treatment caused a significant general decrease in staining on the entire hemi-brain, and significant decrease in the hippocampus. Other areas that were compared did not show significant decreases compared to drug naïve rats, although there was a general pattern of lower GABA binding in anterior cingulate, caudate, NAc, thalamus, and amygdala (Armstrong and Noguchi, 2004). Perhaps an increase in sample size to more than 8 rats in controls, and 7 rats in the methamphetamine treated group may have shown significant decreased [^3^H]-Flunitrazepam binding in these areas.

More recent studies have found that methamphetamine sensitization in mice (4 injections of 2 mg/kg, every other day) caused memory impairment, and autoradiography showed decreased [^3^H]MK-801 binding to NMDA receptors in the cortex, and hippocampus (Lee et al., 2011). Whether NMDA receptors that were lost were expressed on pyramidal or non-pyramidal neurons within the PFC is unclear, and the interval between last methamphetamine exposure and autoradiography was only 24 h, immediately after the passive avoidance test (Lee et al., 2011).

A different mouse sensitization study (1 mg/kg every day for 7 days) produced cognitive deficits that were ameliorated by the GABA_B_ receptor agonist baclofen (1–2 mg/kg acute dose) (Arai et al., 2009). Baclofen has also been shown to ameliorate methamphetamine induced pre-pulse inhibiton and object recognition memory deficits in mice (Mizoguchi and Yamada, 2011). Amphetamine induced dopamine release in the PFC is reduced by baclofen and SKF97541, which activate presynaptic GABA_B_ receptors on dopamine terminals (Balla et al., 2009).

A recent magnetic resonance spectroscopy study in methamphetamine sensitized rat brain dissections (2.5 mg/kg twice per day over 7 days) showed decreased GABA, glutamate, and glutamine levels in the PFC (Bu et al., 2013). Acute methamphetamine administration showed the ability to sequester glutamate in the cortex for over 28 h through upregulation of VGLUT1 (Mark et al., 2007). The Bu et al. study sacrificed the animals within 30 min of the last dose of methamphetamine and one might have expected to find increased glutamate levels in the prefrontal cortex. However, these authors suggest that the reduction in glutamate is likely due to higher demand, while the reduction in GABA was thought to be due both reduced glutamate as a substrate to form GABA, as well as increased GABA metabolism to succinic acid semialdehyde (which was found to be increased) (Bu et al., 2013).

Even though there is vast methodological variation in the above studies, each have shown evidence of cortical GABAergic deficit as a result of methamphetamine treatment. Damage to cortical GABAergic function could account for the cognitive impairments and persistent psychosis symptoms in MAP. The mechanism for this deficit is unknown. The present review proposes that there may be particular vulnerability of GABAergic interneurons, that might contribute to these observations of GABA dysfunction in the cortex as a result of methamphetamine exposure.

## 6. Cortical GABAergic interneuron vulnerability

The NMDA receptor is an ionotropic glutamate receptor that consists of a heterotetramer of two NR1 and two NR2 subunits, and mediates excitatory post-synaptic potentials (Dingledine et al., 1999). NMDA receptors are distributed widely throughout the nervous system (Petralia et al., 1994b).

The downstream effect of NMDA receptor function is highly dependent on the location of the receptors. The expression of *N*-methyl-*D*-aspartate (NMDA) receptors on postsynaptic pyramidal neurons leads to activation of the glutamatergic system and downstream neuronal activation. The expression of NMDA receptors on GABAergic interneurons results in a release of GABA, and has a downstream inhibitory effect. Thus, activation of NMDA receptors on pyramidal neurons leads to further release of glutamate, thereby providing positive feedback; while activation of NMDA receptors on interneurons leads to GABA release and regulation of glutamatergic function.

**Figure 4:**
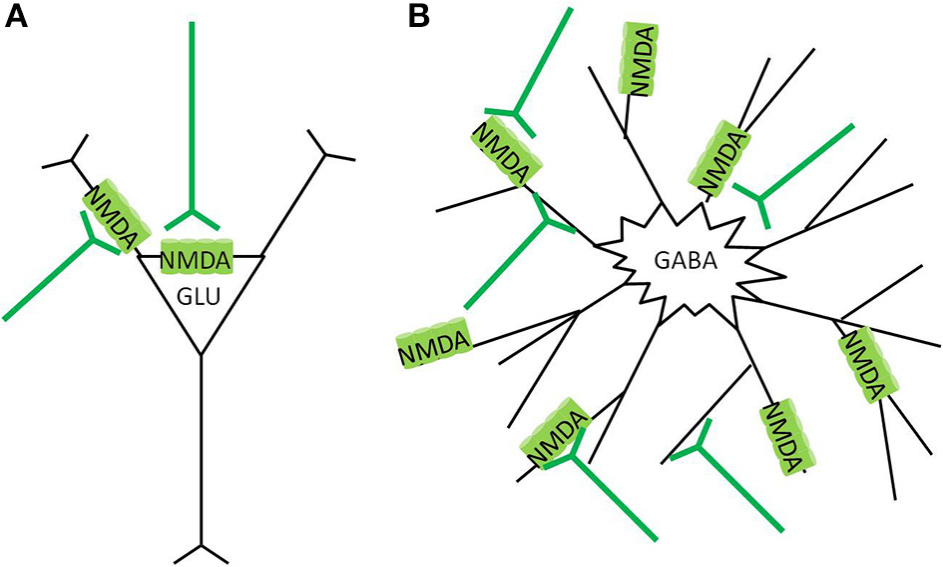
**NMDA receptor localization in the cortex. (A)** Glutamatergic pyramidal neurons express NMDAreceptors preferentially in the post synaptic density (PSD) region, around the soma, and apical dendrites. Glutamate stimulation causes more focused calcium ion influx, and the cell has lower vulnerability to neurotoxicity **(B)** GABAergic interneurons express NMDA receptors more diffusely over the neuron, often at extrasynaptic locations. Glutamate stimulation causes widespread calcium ion influx, and the cell has greater vulnerability to neurotoxicity.

Pyramidal neurons in the cortex primarily express NMDA receptors at the somatic membrane, and proximal dendrites and in post synaptic densities (Aoki et al., 1994; Petralia et al., 1994a), with decreased expression at more distal dendrites (Huntley et al., 1994). Cortical GABAergic interneurons have typically multipolar aspiny dendrites (Kawaguchi, 1995; DeFelipe, 2001), with the majority of NMDA receptors expressed on the dendrites, not close to obvious post synaptic densities (Goldberg et al., 2003a,b) (Figure 4). Dense patterns of NMDAR1 staining appear particularly in the neuropil of layer II and III in the cortex (Aoki et al., 1994; Huntley et al., 1994), which coincide with the dense dendritic projections of basket, chandelier, and double bouquet type interneurons in this region (Benes and Berretta, 2001; DeFelipe, 2001; Gentet, 2012). Discussions of the various types, morphology, and physiological characterizations of GABAergic interneurons are interesting but not within the scope of this paper, and have been have been thoroughly reviewed elsewhere (Benes and Berretta, 2001; DeFelipe, 2001; Gentet, 2012).

The predominance of synaptic NMDA receptors on the pyramidal neurons, and extrasynaptic NMDA receptors on GABAergic interneurons differentiates their vulnerability to neurotoxicity (Papadia and Hardingham, 2007). Synaptic and extrasynaptic NMDA receptors have opposite effects regarding cAMP response element binding protein (CREB) function, gene regulation, and neuron survival (Hardingham et al., 2002). Calcium ion entry through synaptic NMDA receptors induces CREB activity and brain derived neurotrophic factor (BDNF) gene expression as strongly as those caused by direct stimulation of L-type calcium channels. In contrast, calcium ion entry through extrasynaptic NMDA receptors, triggered by bath glutamate exposure or hypoxic conditions, activates a dominant pathway that blocks CREB and thus BDNF expression. In essence, synaptic NMDA receptors, mostly localized on glutamatergic cells, have anti-apoptotic activity, whereas stimulation of extrasynaptic NMDA receptors on interneurons results in glutamate-induced excitotoxicity (Hardingham et al., 2002). Similar CREB inhibiting effects have been found by activation of extrasynaptic NMDA receptors in co-cultured cortical glutamatergic and striatal GABAergic neurons, while synaptic NMDA activation on GABAergic striatal neurons was neuroprotective (Kaufman et al., 2012). Details of the mechanisms of the dicotomous effects of synaptic vs. extrasynaptic NMDA receptor activation is reviewed elsewhere (Papadia and Hardingham, 2007; Hardingham and Bading, 2010). Essentially, lower levels and specific activation of synaptic NMDA receptors promote neuroprotection and inhibits apoptotic pathways; while bath stimulation or over spill from high level stimulation cause extrasynaptic NMDA receptor activation, which not only inhibits CREB and BDNF expression, but also promotes a variety of other pro-death cascades (Papadia and Hardingham, 2007; Hardingham and Bading, 2010; Petralia, 2012).

Activation of predominantly extrasynaptic NMDA receptors on GABAergic interneurons would make these cells more vulnerable to neurotoxicity than pyramidal neurons. Thus, persistent glutamate overflow from the overstimulated thalamus and NAc to the cortex from repeated methamphetamine administration might lead to accelerated damage to interneurons compared to pyramidal neurons. Indeed, an escalating dose multiple binge regimen of methamphetamine administration in rats found that calbindin stained cortical interneurons were more sensitive to methamphetamine toxicity than their NeuN stained pyramidal neurons, as the interneurons were significantly lost from 3 days after the last methamphetamine binge dose, whereas the pyramidal neurons were lost only 30 days after the last dose (Kuczenski et al., 2007). Damage to cortical interneurons would diminish the negative feedback on pyramidal neurons, and result in increased subsequent pyramidal firing.

Cortical GABAergic interneurons synchronize cortical processes (Jones, 1993; Benes and Berretta, 2001). Damage to these neurons, whether by methamphetamine induced neurotoxicity, developmental defect, or other mechanisms would dysregulate glutamate signaling in the cortex. Even transient inhibition of NMDA receptors by a dose of MK-801 that impaired working memory in rats decreased organized bursting activity and increased the number of irregularly discharged single spikes from prefrontal pyramidal neurons (Jackson et al., 2004). Aberrant glutamate signals transmitted through the cortico-striatal-thalamo-cortical loop may result in aberrant behaviors, and psychosis.

## 7. Summary

**Figure 5:**
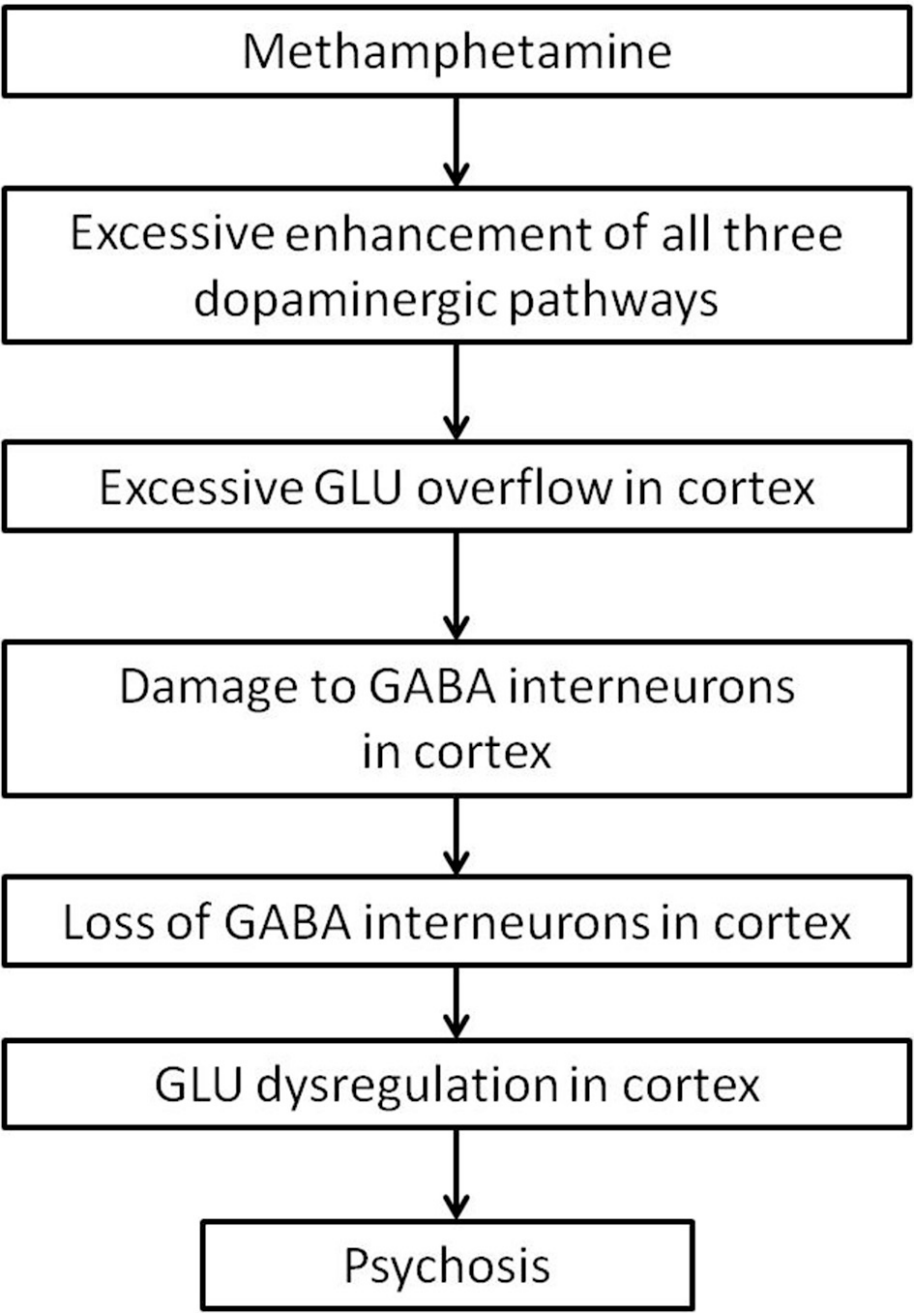
**Summary flow diagram**.

The development of MAP might ultimately be due to damage of cortical interneurons. Cortical interneurons are likely more vulnerable to neurotoxicity, as they have a higher proportion of extrasynaptically expressed NMDA receptors. Methamphetamine use causes an overflow of dopamine in the striatum, which leads to excessive glutamate release into the cortex. Excess glutamate in the cortex might, over time, cause damage to cortical interneurons. Damage to cortical interneurons dysregulate thalamocortical signals, and might result in the presentation of psychotic symptoms as seen in schizophrenia (Figure 5).

Studies of GABAergic disturbance in the cortex as a result of methamphetamine have been presented, however further studies that directly compare cell type vulnerabilities to synaptic or extrasynaptic stimulation, as well as measurements of GABA concentrations, receptor expression and sensitivity in the cortex as a result of methamphetamine sensitization or psychosis, would be required to test this hypothesis.

### Conflict of interest statement

The authors declare that the research was conducted in the absence of any commercial or financial relationships that could be construed as a potential conflict of interest.

## References

[B1] Albin R. L. Makowiec R. L. Hollingsworth Z. R. Dure L. S. 4th. Penney J. B. Young A. B. (1992). Excitatory amino acid binding sites in the basal ganglia of the rat: a quantitative autoradiographic study. Neuroscience 46, 35–48 10.1016/0306-4522(92)90006-N 1317515

[B2] Alexander G. E. Crutcher M. D. (1990). Functional architecture of basal ganglia circuits: neural substrates of parallel processing. Trends Neurosci. 13, 266–271 10.1016/0166-2236(90)90107-L 1695401

[B3] Altar C. A. Hauser K. (1987). Topography of substantia nigra innervation by d1 receptor-containing striatal neurons. Brain Res. 410, 1–11 10.1016/S0006-8993(87)80014-8 2953407

[B4] American Psychiatric Association. (2013). DSM 5. Washington, DC: American Psychiatric Association

[B5] Angrist B. M. Gershon S. (1970). The phenomenology of experimentally induced amphetamine psychosis–preliminary observations. Biol. Psychiatry 2, 95–107 5459137

[B6] Aoki C. Venkatesan C. Go C. Mong J. A. Dawson T. M. (1994). Cellular and subcellular localization of nmda-r1 subunit immunoreactivity in the visual cortex of adult and neonatal rats. J. Neurosci. 14, 5202–5222 8083731 10.1523/JNEUROSCI.14-09-05202.1994 PMC6577065

[B7] Aoki Y. Orikabe L. Takayanagi Y. Yahata N. Mozue Y. Sudo Y. (2013). Volume reductions in frontopolar and left perisylvian cortices in methamphetamine induced psychosis. Schizophr. Res. 147, 355–361 10.1016/j.schres.2013.04.029 23688384

[B8] Arai S. Takuma K. Mizoguchi H. Ibi D. Nagai T. Kamei H. (2009). Gabab receptor agonist baclofen improves methamphetamine-induced cognitive deficit in mice. Eur. J. Pharmacol. 602, 101–104 10.1016/j.ejphar.2008.10.065 19028488

[B9] Armstrong B. D. Noguchi K. K. (2004). The neurotoxic effects of 3,4-methylenedioxymethamphetamine (mdma) and methamphetamine on serotonin, dopamine, and gaba-ergic terminals: an *in-vitro* autoradiographic study in rats. Neurotoxicology 25, 905–914 10.1016/j.neuro.2004.06.003 15474609

[B10] Balla A. Nattini M. E. Sershen H. Lajtha A. Dunlop D. S. Javitt D. C. (2009). Gabab/nmda receptor interaction in the regulation of extracellular dopamine levels in rodent prefrontal cortex and striatum. Neuropharmacology 56, 915–921 10.1016/j.neuropharm.2009.01.021 19371582 PMC4681299

[B11] Balleine B. W. Delgado M. R. Hikosaka O. (2007). The role of the dorsal striatum in reward and decision-making. J. Neurosci. 27, 8161–8165 10.1523/JNEUROSCI.1554-07.2007 17670959 PMC6673072

[B12] Batki S. L. Harris D. S. (2004). Quantitative drug levels in stimulant psychosis: relationship to symptom severity, catecholamines and hyperkinesia. Am. J. Addict. 13, 461–470 10.1080/10550490490512834 15764424

[B13] Bell D. S. (1965). Comparison of amphetamine psychosis and schizophrenia. Br. J. Psychiatry 111, 701–707 10.1192/bjp.111.477.701 14337419

[B14] Bell D. S. (1973). The experimental reproduction of amphetamine psychosis. Arch. Gen. Psychiatry 29, 35–40 10.1001/archpsyc.1973.04200010020003 4711131

[B15] Bellomo M. Giuffrida R. Palmeri A. Sapienza S. (1998). Excitatory amino acids as neurotransmitters of corticostriatal projections: immunocytochemical evidence in the rat. Arch. Ital. Biol. 136, 215–223 9645311

[B16] Benes F. M. Berretta S. (2001). Gabaergic interneurons: implications for understanding schizophrenia and bipolar disorder. Neuropsychopharmacology 25, 1–27 10.1016/S0893-133X(01)00225-1 11377916

[B17] Bramness J. G. Gundersen O. H. Guterstam J. Rognli E. B. Konstenius M. Loberg E. M. (2012). Amphetamine-induced psychosis–a separate diagnostic entity or primary psychosis triggered in the vulnerable? BMC Psychiatry 12:221 10.1186/1471-244X-12-221 23216941 PMC3554477

[B18] Bu Q. Lv L. Yan G. Deng P. Wang Y. Zhou J. (2013). Nmr-based metabonomic in hippocampus, nucleus accumbens and prefrontal cortex of methamphetamine-sensitized rats. Neurotoxicology 36, 17–23 10.1016/j.neuro.2013.02.007 23462569

[B19] Burns L. (2014). World drug report 2013 by united nations office on drugs and crime new york: United nations, 2013isbn: 978-92-1-056168-6, 151 pp. grey literature. Drug Alcohol Rev. 33, 216–216 10.1111/dar.12110

[B20] Bustamante D. You Z.-B. Castel M.-N. Johansson S. Goiny M. Terenius L. (2002). Effect of single and repeated methamphetamine treatment on neurotransmitter release in substantia nigra and neostriatum of the rat. J. Neurochem. 83, 645–654 10.1046/j.1471-4159.2002.01171.x 12390526

[B21] Cadet J. L. Krasnova I. N. (2009). Molecular bases of methamphetamine-induced neurodegeneration. Int. Rev. Neurobiol. 88, 101–119 10.1016/S0074-7742(09)88005-7 19897076 PMC8247532

[B22] Cartier J. Farabee D. Prendergast M. L. (2006). Methamphetamine use, self-reported violent crime, and recidivism among offenders in california who abuse substances. J. Interpers. Violence 21, 435–445 10.1177/0886260505285724 16501213

[B23] Castner S. A. al Tikriti M. S. Baldwin R. M. Seibyl J. P. Innis R. B. Goldman-Rakic P. S. (2000). Behavioral changes and [123i]ibzm equilibrium spect measurement of amphetamine-induced dopamine release in rhesus monkeys exposed to subchronic amphetamine. Neuropsychopharmacology 22, 4–13 10.1016/S0893-133X(99)00080-9 10633485

[B24] Castner S. A. Goldman-Rakic P. S. (1999). Long-lasting psychotomimetic consequences of repeated low-dose amphetamine exposure in rhesus monkeys. Neuropsychopharmacology 20, 10–28 10.1016/S0893-133X(98)00050-5 9885781

[B25] Castner S. A. Vosler P. S. Goldman-Rakic P. S. (2005). Amphetamine sensitization impairs cognition and reduces dopamine turnover in primate prefrontal cortex. Biol. Psychiatry 57, 743–751 10.1016/j.biopsych.2004.12.019 15820231

[B26] Cepeda C. Levine M. S. (1998). Dopamine and n-methyl-d-aspartate receptor interactions in the neostriatum. Dev. Neurosci. 20, 1–18 10.1159/000017294 9600386

[B27] Chang L. Alicata D. Ernst T. Volkow N. (2007). Structural and metabolic brain changes in the striatum associated with methamphetamine abuse. Addiction 102(Suppl. 1), 16–32 10.1111/j.1360-0443.2006.01782.x 17493050

[B28] Chen C. K. Lin S. K. Sham P. C. Ball D. Loh E. W. Hsiao C. C. (2003). Pre-morbid characteristics and co-morbidity of methamphetamine users with and without psychosis. Psychol. Med. 33, 1407–1414 10.1017/S0033291703008353 14672249

[B29] Chung Y. A. Peterson B. S. Yoon S. J. Cho S. N. Chai S. Jeong J. (2010). *In vivo* evidence for long-term cns toxicity, associated with chronic binge use of methamphetamine. Drug Alcohol Depend. 111, 155–160 10.1016/j.drugalcdep.2010.04.005 20566251

[B30] Colfax G. Guzman R. (2006). Club drugs and hiv infection: a review. Clin. Infect. Dis. 42, 1463–1469 10.1086/503259 16619161

[B31] Connell P. H. (1958). Amphetamine Psychosis. London: Chapman & Hall

[B32] Cornish J. L. Kalivas P. W. (2000). Glutamate transmission in the nucleus accumbens mediates relapse in cocaine addiction. J. Neurosci. 20:RC89 10899176 10.1523/JNEUROSCI.20-15-j0006.2000 PMC6772531

[B33] Cretzmeyer M. Sarrazin M. V. Huber D. L. Block R. I. Hall J. A. (2003). Treatment of methamphetamine abuse: research findings and clinical directions. J. Subst. Abuse Treat. 24, 267–277 10.1016/S0740-5472(03)00028-X 12810148

[B34] Cruickshank C. C. Dyer K. R. (2009). A review of the clinical pharmacology of methamphetamine. Addiction 104, 1085–1099 10.1111/j.1360-0443.2009.02564.x 19426289

[B35] Curran C. Byrappa N. McBride A. (2004). Stimulant psychosis: systematic review. Br. J. Psychiatry 185, 196–204 10.1192/bjp.185.3.196 15339823

[B36] Daskalakis Z. J. Fitzgerald P. B. Christensen B. K. (2007). The role of cortical inhibition in the pathophysiology and treatment of schizophrenia. Brain Res. Rev. 56, 427–442 10.1016/j.brainresrev.2007.09.006 17980435

[B37] Davidson C. Gow A. J. Lee T. H. Ellinwood E. H. (2001). Methamphetamine neurotoxicity: necrotic and apoptotic mechanisms and relevance to human abuse and treatment. Brain Res. Brain Res. Rev. 36, 1–22 10.1016/S0165-0173(01)00054-6 11516769

[B38] DeFelipe J. (2001). Cortical interneurons: from cajal to 2001. Progress Brain Res. 136, 215–238 10.1016/S0079-6123(02)36019-9 12143384

[B39] Deng X. Huang Z. Li X. Li Y. Wang Y. Wu D. (2012). Long-term follow-up of patients treated for psychotic symptoms that persist after stopping illicit drug use. Shanghai Arch. Psychiatry 24, 271–278 10.3969/j.issn.1002-0829.2012.05.004 PMC4198875 25328350

[B40] Dingledine R. Borges K. Bowie D. Traynelis S. F. (1999). The glutamate receptor ion channels. Pharmacol. Rev. 51, 7–61 10049997

[B41] Drevets W. C. Gautier C. Price J. C. Kupfer D. J. Kinahan P. E. Grace A. A. (2001). Amphetamine-induced dopamine release in human ventral striatum correlates with euphoria. Biol. Psychiatry 49, 81–96 10.1016/S0006-3223(00)01038-6 11164755

[B42] Ernst T. Chang L. Leonido-Yee M. Speck O. (2000). Evidence for long-term neurotoxicity associated with methamphetamine abuse: a 1h mrs study. Neurology 54, 1344–1349 10.1212/WNL.54.6.1344 10746608

[B43] Fallon J. H. Moore R. Y. (1978). Catecholamine innervation of the basal forebrain iv. topography of the dopamine projection to the basal forebrain and neostriatum. J. Comp. Neurol. 180, 545–579 10.1002/cne.901800310 659674

[B44] Farrell M. Marsden J. Ali R. Ling W. (2002). Methamphetamine: drug use and psychoses becomes a major public health issue in the asia pacific region. Addiction 97, 771–772 10.1046/j.1360-0443.2002.00195.x 12133111

[B45] Featherstone R. Kapur S. Fletcher P. (2007). The amphetamine-induced sensitized state as a model of schizophrenia. Prog. Neuro-Psychopharmacol. Biol. Psychiatry 31, 1556–1571 10.1016/j.pnpbp.2007.08.025 17884274

[B46] Forrest A. D. Coto C. A. Siegel S. J. (2014). Animal models of psychosis: current state and future directions. Curr. Behav. Neurosci. Rep. 1, 100–116 10.1007/s40473-014-0013-2 PMC4157659 25215267

[B47] Fowler J. S. Volkow N. D. Logan J. Alexoff D. Telang F. Wang G. J. (2008). Fast uptake and long-lasting binding of methamphetamine in the human brain: comparison with cocaine. Neuroimage 43, 756–763 10.1016/j.neuroimage.2008.07.020 18708148 PMC2606665

[B48] Gabernet L. Jadhav S. P. Feldman D. E. Carandini M. Scanziani M. (2005). Somatosensory integration controlled by dynamic thalamocortical feed-forward inhibition. Neuron 48, 315–327 10.1016/j.neuron.2005.09.022 16242411

[B49] Gaspar P. Bloch B. Moine C. (1995). D1 and d2 receptor gene expression in the rat frontal cortex: cellular localization in different classes of efferent neurons. Eur. J. Neurosci. 7, 1050–1063 10.1111/j.1460-9568.1995.tb01092.x 7613610

[B50] Gentet L. J. (2012). Functional diversity of supragranular gabaergic neurons in the barrel cortex. Front. Neural Circuits 6:52 10.3389/fncir.2012.00052 22912602 PMC3421449

[B51] Gerfen C. R. (1989). The neostriatal mosaic: striatal patch-matrix organization is related to cortical lamination. Science 246, 385–388 10.1126/science.2799392 2799392

[B52] Giorgetti M. Hotsenpiller G. Froestl W. Wolf M. (2002). *In vivo* modulation of ventral tegmental area dopamine and glutamate efflux by local gaba_b_ receptors is altered after repeated amphetamine treatment. Neuroscience 109, 585–595 10.1016/S0306-4522(01)00510-3 11823068

[B53] Gold J. M. (2004). Cognitive deficits as treatment targets in schizophrenia. Schizophr. Res. 72, 21–28 10.1016/j.schres.2004.09.008 15531404

[B54] Goldberg J. H. Tamas G. Aronov D. Yuste R. (2003a). Calcium microdomains in aspiny dendrites. Neuron 40, 807–821 10.1016/S0896-6273(03)00714-1 14622584

[B55] Goldberg J. H. Yuste R. Tamas G. (2003b). Ca2+ imaging of mouse neocortical interneurone dendrites: contribution of ca2+-permeable ampa and nmda receptors to subthreshold ca2+dynamics. J. Physiol. 551(pt 1), 67–78 10.1113/jphysiol.2003.042598 12844507 PMC2343145

[B56] Goldman-Rakic P. S. (1994). Working memory dysfunction in schizophrenia. J. Neuropsychiatry Clin. Neurosci. 6, 348–357 7841806 10.1176/jnp.6.4.348

[B57] Goldman-Rakic P. S. (1995). Cellular basis of working memory. Neuron 14, 477–485 10.1016/0896-6273(95)90304-6 7695894

[B58] Goldman-Rakic P. S. Castner S. A. Svensson T. H. Siever L. J. Williams G. V. (2004). Targeting the dopamine d1 receptor in schizophrenia: insights for cognitive dysfunction. Psychopharmacology 174, 3–16 10.1007/s00213-004-1793-y 15118803

[B59] Goldman-Rakic P. S. Leranth C. Williams S. M. Mons N. Geffard M. (1989). Dopamine synaptic complex with pyramidal neurons in primate cerebral cortex. Proc. Natl. Acad. Sci. U.S.A. 86, 9015–9019 10.1073/pnas.86.22.9015 2573073 PMC298423

[B60] Gonon F. (1988). Nonlinear relationship between impulse flow and dopamine released by rat midbrain dopaminergic neurons as studied by *in vivo* electrochemistry. Neuroscience 24, 19–28 10.1016/0306-4522(88)90307-7 3368048

[B61] Graveland G. A. Williams R. S. DiFiglia M. (1985). A golgi study of the human neostriatum: neurons and afferent fibers. J. Comp. Neurol. 234, 317–333 10.1002/cne.902340304 3988987

[B62] Green A. I. Halkitis P. N. (2006). Crystal methamphetamine and sexual sociality in an urban gay subculture: an elective affinity. Cult. Health Sex. 8, 317–333 10.1080/13691050600783320 16846941

[B63] Green M. F. (1996). What are the functional consequences of neurocognitive deficits in schizophrenia? Am. J. Psychiatry 153, 321–330 8610818 10.1176/ajp.153.3.321

[B64] Haber S. N. Knutson B. (2010). The reward circuit: linking primate anatomy and human imaging. Neuropsychopharmacology 35, 4–26 10.1038/npp.2009.129 19812543 PMC3055449

[B65] Haber S. N. McFarland N. R. (1999). The concept of the ventral striatum in nonhuman primates. Ann. N.Y. Acad. Sci. 877, 33–48 10.1111/j.1749-6632.1999.tb09259.x 10415641

[B66] Halkitis P. N. Parsons J. T. Stirratt M. J. (2001). A double epidemic: crystal methamphetamine drug use in relation to hiv transmission among gay men. J. Homosexual. 41, 17–35 10.1300/J082v41n02_02 11482426

[B67] Hardingham G. E. Bading H. (2010). Synaptic versus extrasynaptic nmda receptor signalling: implications for neurodegenerative disorders. Nat. Rev. Neurosci. 11, 682–696 10.1038/nrn2911 20842175 PMC2948541

[B68] Hardingham G. E. Fukunaga Y. Bading H. (2002). Extrasynaptic nmdars oppose synaptic nmdars by triggering creb shut-off and cell death pathways. Nat. Neurosci. 5, 405–414 10.1038/nn835 11953750

[B69] Homayoun H. Moghaddam B. (2006). Progression of cellular adaptations in medial prefrontal and orbitofrontal cortex in response to repeated amphetamine. J. Neurosci. 26, 8025–8039 10.1523/JNEUROSCI.0842-06.2006 16885216 PMC2954613

[B70] Horan W. P. Green M. F. Knowlton B. J. Wynn J. K. Mintz J. Nuechterlein K. H. (2008). Impaired implicit learning in schizophrenia. Neuropsychology 22, 606–617 10.1037/a0012602 18763880 PMC2548320

[B71] Howells F. M. Uhlmann A. Temmingh H. Sinclair H. Meintjes E. Wilson D. (2014). ^1^h-magnetic resonance spectroscopy (^1^ h-mrs) in methamphetamine dependence and methamphetamine induced psychosis. Schizophr. Res. 153, 122–128 10.1016/j.schres.2014.01.029 24529366

[B72] Hu X. T. White F. J. (1997). Dopamine enhances glutamate-induced excitation of rat striatal neurons by cooperative activation of d1 and d2 class receptors. Neurosci. Lett. 224, 61–65 10.1016/S0304-3940(97)13443-7 9132692

[B73] Huntley G. W. Vickers J. Janssen W. Brose N. Heinemann S. Morrison J. (1994). Distribution and synaptic localization of immunocytochemically identified nmda receptor subunit proteins in sensory-motor and visual cortices of monkey and human. J. Neurosci. 14, 3603–3619 8207475 10.1523/JNEUROSCI.14-06-03603.1994 PMC6576922

[B74] Iwanami A. Kato N. Nakatani Y. (1991). P300 in methamphetamine psychosis. Biol. Psychiatry 30, 726–730 10.1016/0006-3223(91)90018-H 1958770

[B75] Iwanami A. Kuroki N. Iritani S. Isono H. Okajima Y. Kamijima K. (1998). P3a of event-related potential in chronic methamphetamine dependence. J. Nerv. Ment. Dis. 186, 746–751 10.1097/00005053-199812000-00002 9865812

[B76] Iwanami A. Suga I. Kato N. Nakatani Y. Kaneko T. (1993). Event-related potentials in methamphetamine psychosis during an auditory discrimination task. a preliminary report. Eur. Arch. Psychiatry Clin. Neurosci. 242, 203–208 10.1007/BF02189964 8096397

[B77] Iwanami A. Sugiyama A. Kuroki N. Toda S. Kato N. Nakatani Y. (1994). Patients with methamphetamine psychosis admitted to a psychiatric hospital in japan. a preliminary report. Acta Psychiat. Scand. 89, 428–432 10.1111/j.1600-0447.1994.tb01541.x 8085475

[B78] Iyo M. Namba H. Yanagisawa M. Hirai S. Yui N. Fukui S. (1997). Abnormal cerebral perfusion in chronic methamphetamine abusers: a study using 99mtc-hmpao and spect. Prog. Neuro-psychopharmacol. Biol. Psychiatry 21, 789–796 10.1016/S0278-5846(97)00079-1 9278950

[B79] Jackson M. E. Homayoun H. Moghaddam B. (2004). Nmda receptor hypofunction produces concomitant firing rate potentiation and burst activity reduction in the prefrontal cortex. Proc. Natl. Acad. Sci. U.S.A. 101, 8467–8472 10.1073/pnas.0308455101 15159546 PMC420417

[B80] Jacobs E. Fujii D. Schiffman J. Bello I. (2008). An exploratory analysis of neurocognition in methamphetamine-induced psychotic disorder and paranoid schizophrenia. Cogn. Behav. Neurol. 21, 98–103 10.1097/WNN.0b013e31816bdf90 18541986

[B81] Janowsky D. S. Risch C. (1979). Amphetamine psychosis and psychotic symptoms. Psychopharmacology 65, 73–77 10.1007/BF00491982 116294

[B82] Jones E. G. (1993). Gabaergic neurons and their role in cortical plasticity in primates. Cereb. Cortex 3, 361–372 10.1093/cercor/3.5.361-a 8260806

[B83] Kalivas P. W. Volkow N. D. (2005). The neural basis of addiction: a pathology of motivation and choice. Am. J. Psychiatry 162, 1403–1413 10.1176/appi.ajp.162.8.1403 16055761

[B84] Kamei H. Nagai T. Nakano H. Togan Y. Takayanagi M. Takahashi K. (2006). Repeated methamphetamine treatment impairs recognition memory through a failure of novelty-induced erk1/2 activation in the prefrontal cortex of mice. Biol. Psychiatry 59, 75–84 10.1016/j.biopsych.2005.06.006 16139811

[B85] Kankaanpää A. Meririnne E. Lillsunde P. Seppälä T. (1998). The acute effects of amphetamine derivatives on extracellular serotonin and dopamine levels in rat nucleus accumbens. Pharmacol. Biochem. Behav. 59, 1003–1009 10.1016/S0091-3057(97)00527-3 9586861

[B86] Kaufman A. M. Milnerwood A. J. Sepers M. D. Coquinco A. She K. Wang L. (2012). Opposing roles of synaptic and extrasynaptic nmda receptor signaling in cocultured striatal and cortical neurons. J. Neurosci. 32, 3992–4003 10.1523/JNEUROSCI.4129-11.2012 22442066 PMC6621208

[B87] Kawaguchi Y. (1995). Physiological subgroups of nonpyramidal cells with specific morphological characteristics in layer ii/iii of rat frontal cortex. J. Neurosci. 15, 2638–2655 7722619 10.1523/JNEUROSCI.15-04-02638.1995 PMC6577784

[B88] Kemp J. M. Powell T. P. (1970). The cortico-striate projection in the monkey. Brain J. Neurol. 93, 525–546 10.1093/brain/93.3.525 4990231

[B89] Kittirattanapaiboon P. Mahatnirunkul S. Booncharoen H. Thummawomg P. Dumrongchai U. Chutha W. (2010). Long-term outcomes in methamphetamine psychosis patients after first hospitalisation. Drug Alcohol Rev. 29, 456–461 10.1111/j.1465-3362.2010.00196.x 20636664

[B90] Koob G. F. Bloom F. E. (1988). Cellular and molecular mechanisms of drug dependence. Science 242, 715–723 10.1126/science.2903550 2903550

[B91] Kuczenski R. Everall I. P. Crews L. Adame A. Grant I. Masliah E. (2007). Escalating dose-multiple binge methamphetamine exposure results in degeneration of the neocortex and limbic system in the rat. Exp. Neurol. 207, 42–51 10.1016/j.expneurol.2007.05.023 17603040 PMC2796472

[B92] Langford D. Adame A. Grigorian A. Grant I. McCutchan J. A. Ellis R. J. (2003). Patterns of selective neuronal damage in methamphetamine-user aids patients. J. Acquir. Immune Defic. Syndr. 34, 467–474 10.1097/00126334-200312150-00004 14657756

[B93] Lee K. W. Kim H. C. Lee S. Y. Jang C. G. (2011). Methamphetamine-sensitized mice are accompanied by memory impairment and reduction of n-methyl-d-aspartate receptor ligand binding in the prefrontal cortex and hippocampus. Neuroscience 178, 101–107 10.1016/j.neuroscience.2011.01.025 21256196

[B94] Lewis D. A. Curley A. A. Glausier J. R. Volk D. W. (2012). Cortical parvalbumin interneurons and cognitive dysfunction in schizophrenia. Trends Neurosci. 35, 57–67 10.1016/j.tins.2011.10.004 22154068 PMC3253230

[B95] Lewis D. A. Volk D. W. Hashimoto T. (2004). Selective alterations in prefrontal cortical gaba neurotransmission in schizophrenia: a novel target for the treatment of working memory dysfunction. Psychopharmacology 174, 143–150 10.1007/s00213-003-1673-x 15205885

[B96] Liu X. Chang L. Vigorito M. Kass M. Li H. Chang S. L. (2009). Methamphetamine-induced behavioral sensitization is enhanced in the hiv-1 transgenic rat. J. Neuroimmune Pharmacol. 4, 309–316 10.1007/s11481-009-9160-8 19444617

[B97] Machiyama Y. (1992). Chronic methamphetamine intoxication model of schizophrenia in animals. Schizophr. Bull. 18, 107–113 10.1093/schbul/18.1.107 1553490

[B98] Mahoney J. J. Kalechstein A. D. Garza R. D. L. Newton T. F. (2008). Presence and persistence of psychotic symptoms in cocaine- versus methamphetamine-dependent participants. Am. J. Addict. 17, 83–98 10.1080/10550490701861201 18393050 PMC4119877

[B99] Mark K. A. Quinton M. S. Russek S. J. Yamamoto B. K. (2007). Dynamic changes in vesicular glutamate transporter 1 function and expression related to methamphetamine-induced glutamate release. J. Neurosci. 27, 6823–6831 10.1523/JNEUROSCI.0013-07.2007 17581970 PMC6672707

[B100] Mark K. A. Soghomonian J. J. Yamamoto B. K. (2004). High-dose methamphetamine acutely activates the striatonigral pathway to increase striatal glutamate and mediate long-term dopamine toxicity. J. Neurosci. 24, 11449–11456 10.1523/JNEUROSCI.3597-04.2004 15601951 PMC6730359

[B101] Maurice N. Deniau J.-M. Glowinski J. Thierry A.-M. (1999). Relationships between the prefrontal cortex and the basal ganglia in the rat: physiology of the cortico-nigral circuits. J. Neurosci. 19, 4674–4681 10341265 10.1523/JNEUROSCI.19-11-04674.1999 PMC6782607

[B102] McKetin R. Hickey K. Devlin K. Lawrence K. (2010). The risk of psychotic symptoms associated with recreational methamphetamine use. Drug Alcohol Rev. 29, 358–363 10.1111/j.1465-3362.2009.00160.x 20636650

[B103] McKetin R. McLaren J. Lubman D. I. Hides L. (2006). The prevalence of psychotic symptoms among methamphetamine users. Addiction 101, 1473–1478 10.1111/j.1360-0443.2006.01496.x 16968349

[B104] Medhus S. Mordal J. Holm B. Mørland J. Bramness J. G. (2013). A comparison of symptoms and drug use between patients with methamphetamine associated psychoses and patients diagnosed with schizophrenia in two acute psychiatric wards. Psychiatry Res. 206, 17–21 10.1016/j.psychres.2012.09.023 23036490

[B105] Mizoguchi H. Yamada K. (2011). Pharmacologic treatment with gabab receptor agonist of methamphetamine-induced cognitive impairment in mice. Curr. Neuropharmacol. 9, 109 10.2174/157015911795016976 21886573 PMC3137162

[B106] Mogenson G. J. Jones D. L. Yim C. Y. (1980). From motivation to action: functional interface between the limbic system and the motor system. Prog. Neurobiol. 14, 69–97 10.1016/0301-0082(80)90018-0 6999537

[B107] Muly E. C. Szigeti K. Goldman-Rakic P. S. (1998). D1 receptor in interneurons of macaque prefrontal cortex: distribution and subcellular localization. J. Neurosci. 18, 10553–10565 9852592 10.1523/JNEUROSCI.18-24-10553.1998 PMC6793362

[B108] Nagai T. Takuma K. Dohniwa M. Ibi D. Mizoguchi H. Kamei H. (2007). Repeated methamphetamine treatment impairs spatial working memory in rats: reversal by clozapine but not haloperidol. Psychopharmacology 194, 21–32 10.1007/s00213-007-0820-1 17514479

[B109] Nagai T. Yamada K. (2010). Molecular mechanism for methamphetamine-induced memory impairment. Nihon Arukoru Yakubutsu Igakkai zasshi 45, 81–91 20486560

[B110] Nicola S. M. (2007). The nucleus accumbens as part of a basal ganglia action selection circuit. Psychopharmacology 191, 521–550 10.1007/s00213-006-0510-4 16983543

[B111] Nordahl T. E. Salo R. Possin K. Gibson D. R. Flynn N. Leamon M. (2002). Low n-acetyl-aspartate and high choline in the anterior cingulum of recently abstinent methamphetamine-dependent subjects: a preliminary proton mrs study. magnetic resonance spectroscopy. Psychiatry Res. 116, 43–52 10.1016/S0925-4927(02)00088-4 12426033

[B112] Padgett C. L. Lalive A. L. Tan K. R. Terunuma M. Munoz M. B. Pangalos M. N. (2012). Methamphetamine-evoked depression of gaba(b) receptor signaling in gaba neurons of the vta. Neuron 73, 978–989 10.1016/j.neuron.2011.12.031 22405207 PMC3560416

[B113] Panenka W. J. Procyshyn R. M. Lecomte T. MacEwan G. W. Flynn S. W. Honer W. G. (2013). Methamphetamine use: a comprehensive review of molecular, preclinical and clinical findings. Drug Alcohol Depend. 129, 167–179 10.1016/j.drugalcdep.2012.11.016 23273775

[B114] Papadia S. Hardingham G. E. (2007). The dichotomy of nmda receptor signaling. Neuroscientist 13, 572–579 10.1177/10738584070130060401 18000068 PMC2830536

[B115] Paulson P. E. Robinson T. E. (1995). Amphetamine-induced time-dependent sensitization of dopamine neurotransmission in the dorsal and ventral striatum: a microdialysis study in behaving rats. Synapse 19, 56–65 10.1002/syn.890190108 7709344 PMC1859849

[B116] Petralia R. Yokotani N. Wenthold R. (1994a). Light and electron microscope distribution of the nmda receptor subunit nmdar1 in the rat nervous system using a selective anti-peptide antibody. J. Neurosci. 14, 667–696 8301357 10.1523/JNEUROSCI.14-02-00667.1994 PMC6576818

[B117] Petralia R. S. Wang Y. X. Wenthold R. J. (1994b). The nmda receptor subunits nr2a and nr2b show histological and ultrastructural localization patterns similar to those of nr1. J. Neurosci. 14, 6102–6120 7931566 10.1523/JNEUROSCI.14-10-06102.1994 PMC6576991

[B118] Petralia R. S. (2012). Distribution of extrasynaptic NMDA receptors on neurons. ScientificWorldJournal 2012:267120 10.1100/2012/267120 22654580 PMC3361219

[B119] Plüddemann A. Flisher A. J. McKetin R. Parry C. Lombard C. (2010). Methamphetamine use, aggressive behavior and other mental health issues among high-school students in cape town, south africa. Drug Alcohol Depend. 109, 14–19 10.1016/j.drugalcdep.2009.11.021 20064699 PMC3784347

[B120] Reiner B. C. Keblesh J. P. Xiong H. (2009). Methamphetamine abuse, hiv infection, and neurotoxicity. Int. J. Physiol. Pathophysiol. Pharmacol. 1, 162–179 20411028 PMC2856939

[B121] Reubi J. C. Iversen L. L. Jessell T. M. (1977). Dopamine selectively increases 3h-gaba release from slices of rat substantia nigra *in vitro*. Nature 268, 652–654 10.1038/268652a0 197423

[B122] Reubi J. C. Iversen L. L. Jessell T. M. (1978). Regulation of gaba release by dopamine in the rat substantia nigra. Adv. Biochem. Psychopharmacol. 19, 401–404 696468

[B123] Robinson T. E. Becker J. B. (1986). Enduring changes in brain and behavior produced by chronic amphetamine administration: a review and evaluation of animal models of amphetamine psychosis. Brain Res. 396, 157–198 10.1016/0165-0173(86)90002-0 3527341

[B124] Robinson T. E. Kolb B. (1997). Persistent structural modifications in nucleus accumbens and prefrontal cortex neurons produced by previous experience with amphetamine. J. Neurosci. 17, 8491–8497 9334421 10.1523/JNEUROSCI.17-21-08491.1997 PMC6573726

[B125] Robinson T. E. Kolb B. (1999). Alterations in the morphology of dendrites and dendritic spines in the nucleus accumbens and prefrontal cortex following repeated treatment with amphetamine or cocaine. Eur. J. Neurosci. 11, 1598–1604 10.1046/j.1460-9568.1999.00576.x 10215912

[B126] Rymar V. V. Sasseville R. Luk K. C. Sadikot A. F. (2004). Neurogenesis and stereological morphometry of calretinin-immunoreactive gabaergic interneurons of the neostriatum. J. Comp. Neurol. 469, 325–339 10.1002/cne.11008 14730585

[B127] Salamone J. D. Correa M. Farrar A. Mingote S. M. (2007). Effort-related functions of nucleus accumbens dopamine and associated forebrain circuits. Psychopharmacology 191, 461–482 10.1007/s00213-006-0668-9 17225164

[B128] Sato M. Numachi Y. Hamamura T. (1992). Relapse of paranoid psychotic state in methamphetamine model of schizophrenia. Schizophr. Bull. 18, 115–122 10.1093/schbul/18.1.115 1553491

[B129] Schroder N. O'Dell S. J. Marshall J. F. (2003). Neurotoxic methamphetamine regimen severely impairs recognition memory in rats. Synapse 49, 89–96 10.1002/syn.10210 12740864

[B130] Scott J. C. Woods S. P. Matt G. E. Meyer R. A. Heaton R. K. Atkinson J. H. (2007). Neurocognitive effects of methamphetamine: a critical review and meta-analysis. Neuropsychol. Rev. 17, 275–297 10.1007/s11065-007-9031-0 17694436

[B131] Segal D. S. Kuczenski R. (1997). An escalating dose binge model of amphetamine psychosis: behavioral and neurochemical characteristics. J. Neurosci. 17, 2551–2566 9065515 10.1523/JNEUROSCI.17-07-02551.1997 PMC6573483

[B132] Segal D. S. Kuczenski R. (2001). Escalating dose-binge exposure to amphetamine and methamphetamine: behavioral and neurochemical characterization, in Contemporary Neuropsychiatry, eds Miyoshi K. Shapiro C. Gaviria M. Morita Y. (Springer), 330–335

[B133] Sekine Y. Minabe Y. Kawai M. Suzuki K. Iyo M. Isoda H. (2002). Metabolite alterations in basal ganglia associated with methamphetamine-related psychiatric symptoms. a proton mrs study. Neuropsychopharmacology 27, 453–461 10.1016/S0893-133X(02)00321-4 12225702

[B134] Selemon L. D. Begovic A. Goldman-Rakic P. S. Castner S. A. (2007). Amphetamine sensitization alters dendritic morphology in prefrontal cortical pyramidal neurons in the non-human primate. Neuropsychopharmacology 32, 919–931 10.1038/sj.npp.1301179 16936713

[B135] Silber B. Y. Croft R. J. Papafotiou K. Stough C. (2006). The acute effects of d-amphetamine and methamphetamine on attention and psychomotor performance. Psychopharmacology 187, 154–169 10.1007/s00213-006-0410-7 16761129

[B136] Simon H. Scatton B. Le Moal M. (1980). Dopaminergic A10 neurones are involved in cognitive functions. Nature 286, 150–151 10.1038/286150a0 7402306

[B137] Sirinathsinghji D. Dunnett S. Isacson O. Clarke D. Kendrick K. Björklund A. (1988). Striatal grafts in rats with unilateral neostriatal lesionsâĂŤii.s *in vivo* monitoring of gaba release in globus pallidus and substantia nigra. Neuroscience 24, 803–811 10.1016/0306-4522(88)90068-1 3380300

[B138] Sora I. Li B. Fumushima S. Fukui A. Arime Y. Kasahara Y. (2009). Monoamine transporter as a target molecule for psychostimulants. Int. Rev. Neurobiol. 85, 29–33 10.1016/S0074-7742(09)85003-4 19607959

[B139] Srisurapanont M. Ali R. Marsden J. Sunga A. Wada K. Monteiro M. (2003). Psychotic symptoms in methamphetamine psychotic in-patients. Int. J. Neuropsychopharmacol. 6, 347–352 10.1017/S1461145703003675 14604449

[B140] Srisurapanont M. Arunpongpaisal S. Wada K. Marsden J. Ali R. Kongsakon R. (2011). Comparisons of methamphetamine psychotic and schizophrenic symptoms: a differential item functioning analysis. Prog. Neuro-Psychopharmacol. Biol. Psychiatry 35, 959–964 10.1016/j.pnpbp.2011.01.014 21277930

[B141] Stephans S. E. Yamamoto B. K. (1994). Methamphetamine-induced neurotoxicity: roles for glutamate and dopamine efflux. Synapse 17, 203–209 10.1002/syn.890170310 7974204

[B142] Stephans S. E. Yamamoto B. Y. (1995). Effect of repeated methamphetamine administrations on dopamine and glutamate efflux in rat prefrontal cortex. Brain Res. 700, 99–106 10.1016/0006-8993(95)00938-M 8624733

[B143] Sulzer D. Chen T. K. Lau Y. Y. Kristensen H. Rayport S. Ewing A. (1995). Amphetamine redistributes dopamine from synaptic vesicles to the cytosol and promotes reverse transport. J. Neurosci. 15(5 pt 2), 4102–4108 7751968 10.1523/JNEUROSCI.15-05-04102.1995 PMC6578196

[B144] Surmeier D. J. Ding J. Day M. Wang Z. Shen W. (2007). D1 and d2 dopamine-receptor modulation of striatal glutamatergic signaling in striatal medium spiny neurons. Trends Neurosci. 30, 228–235 10.1016/j.tins.2007.03.008 17408758

[B145] Tepper J. M. Bolam J. P. (2004). Functional diversity and specificity of neostriatal interneurons. Curr. Opin. Neurobiol. 14, 685–692 10.1016/j.conb.2004.10.003 15582369

[B146] Timmerman W. Westerink B. H. (1997). Electrical stimulation of the substantia nigra reticulata: detection of neuronal extracellular gaba in the ventromedial thalamus and its regulatory mechanism using microdialysis in awake rats. Synapse 26, 62–71 10.1002/(SICI)1098-2396(199705)26:1<62::AID-SYN7>3.0.CO;2-C 9097406

[B147] Tomiyama G. (1990). Chronic schizophrenia-like states in methamphetamine psychosis. Japan. J. Psychiatry Neurol. 44, 531–539 2074612 10.1111/j.1440-1819.1990.tb01626.x

[B148] Tzschentke T. (2001). Pharmacology and behavioral pharmacology of the mesocortical dopamine system. Prog. Neurobiol. 63, 241–320 10.1016/S0301-0082(00)00033-2 11115727

[B149] Ujike H. Sato M. (2004). Clinical features of sensitization to methamphetamine observed in patients with methamphetamine dependence and psychosis. Ann. N.Y. Acad. Sci. 1025, 279–287 10.1196/annals.1316.035 15542728

[B150] United Nations Office. (2011). World drug report 2011, in Technical Report (Vienna: United Nations Publications).

[B151] Volkow N. D. Chang L. Wang G. J. Fowler J. S. Ding Y. S. Sedler M. (2001a). Low level of brain dopamine d2 receptors in methamphetamine abusers: association with metabolism in the orbitofrontal cortex. Am. J. Psychiatry 158, 2015–2021 10.1176/appi.ajp.158.12.2015 11729018

[B152] Volkow N. D. Chang L. Wang G. J. Fowler J. S. Leonido-Yee M. Franceschi D. (2001b). Association of dopamine transporter reduction with psychomotor impairment in methamphetamine abusers. Am. J. Psychiatry 158, 377–382 10.1176/appi.ajp.158.3.377 11229977

[B153] Volkow N. D. Wang G. J. Fowler J. S. Gatley S. J. Ding Y. S. Logan J. (1996). Relationship between psychostimulant-induced high and dopamine transporter occupancy. Proc. Natl. Acad. Sci. U.S.A. 93, 10388–10392 10.1073/pnas.93.19.10388 8816810 PMC38394

[B154] Weich L. Pienaar W. (2009). Occurrence of comorbid substance use disorders among acute psychiatric inpatients at stikland hospital in the western cape, south africa. Afr. J. Psychiatry 12, 213–217 10.4314/ajpsy.v12i3.48496 19750250

[B155] Williams S. Goldman-Rakic P. (1998). Widespread origin of the primate mesofrontal dopamine system. Cereb. Cortex 8, 321–345 10.1093/cercor/8.4.321 9651129

[B156] Wilson C. J. (1993). The generation of natural firing patterns in neostriatal neurons. Prog. Brain Res. 99, 277–297 10.1016/S0079-6123(08)61352-7 8108553

[B157] Wilson C. J. Kawaguchi Y. (1996). The origins of two-state spontaneous membrane potential fluctuations of neostriatal spiny neurons. J. Neurosci. 16, 2397–2410 8601819 10.1523/JNEUROSCI.16-07-02397.1996 PMC6578540

[B158] Wilson J. M. Kalasinsky K. S. Levey A. I. Bergeron C. Reiber G. Anthony R. M. (1996). Striatal dopamine nerve terminal markers in human, chronic methamphetamine users. Nat. Med. 2, 699–703 10.1038/nm0696-699 8640565

[B159] Xue C.-J. Ng J. P. Li Y. Wolf M. E. (1996). Acute and repeated systemic amphetamine administration: effects on extracellular glutamate, aspartate, and serine levels in rat ventral tegmental area and nucleus accumbens. J. Neurochem. 67, 352–363 10.1046/j.1471-4159.1996.67010352.x 8667013

[B160] Yin H. H. Knowlton B. J. (2006). The role of the basal ganglia in habit formation. Nat. Rev. Neurosci. 7, 464–476 10.1038/nrn1919 16715055

[B161] Yui K. Ishiguro T. Goto K. Ikemoto S. (1997). Precipitating factors in spontaneous recurrence of methamphetamine psychosis. Psychopharmacology 134, 303–308 10.1007/s002130050453 9438680

[B162] Yui K. Ishiguro T. Goto K. Ikemoto S. Kamata Y. (1999). Spontaneous recurrence of methampetamine psychosis: increased sensitivity to stress associated with noradrenergic hyperactivity and dopaminergic change. Eur. Arch. Psychiatry Clin. Neurosci. 249, 103–111 10.1007/s004060050073 10369157

[B163] Zhang Y. Loonam T. M. Noailles P.-A. Angulo J. A. (2001). Comparison of cocaine-and methamphetamine-evoked dopamine and glutamate overflow in somatodendritic and terminal field regions of the rat brain during acute, chronic, and early withdrawal conditions. Ann. N.Y. Acad. Sci. 937, 93–120 10.1111/j.1749-6632.2001.tb03560.x 11458542

